# Supplementary figures and images for: Differential expression of AtWAKL10 in response to nitric oxide suggests a putative role in biotic and abiotic stress responses
Source: PeerJ. 2019 Aug 16;7:e7383. doi: 10.7717/peerj.7383 (PMC6699482; doi:10.7717/peerj.7383)

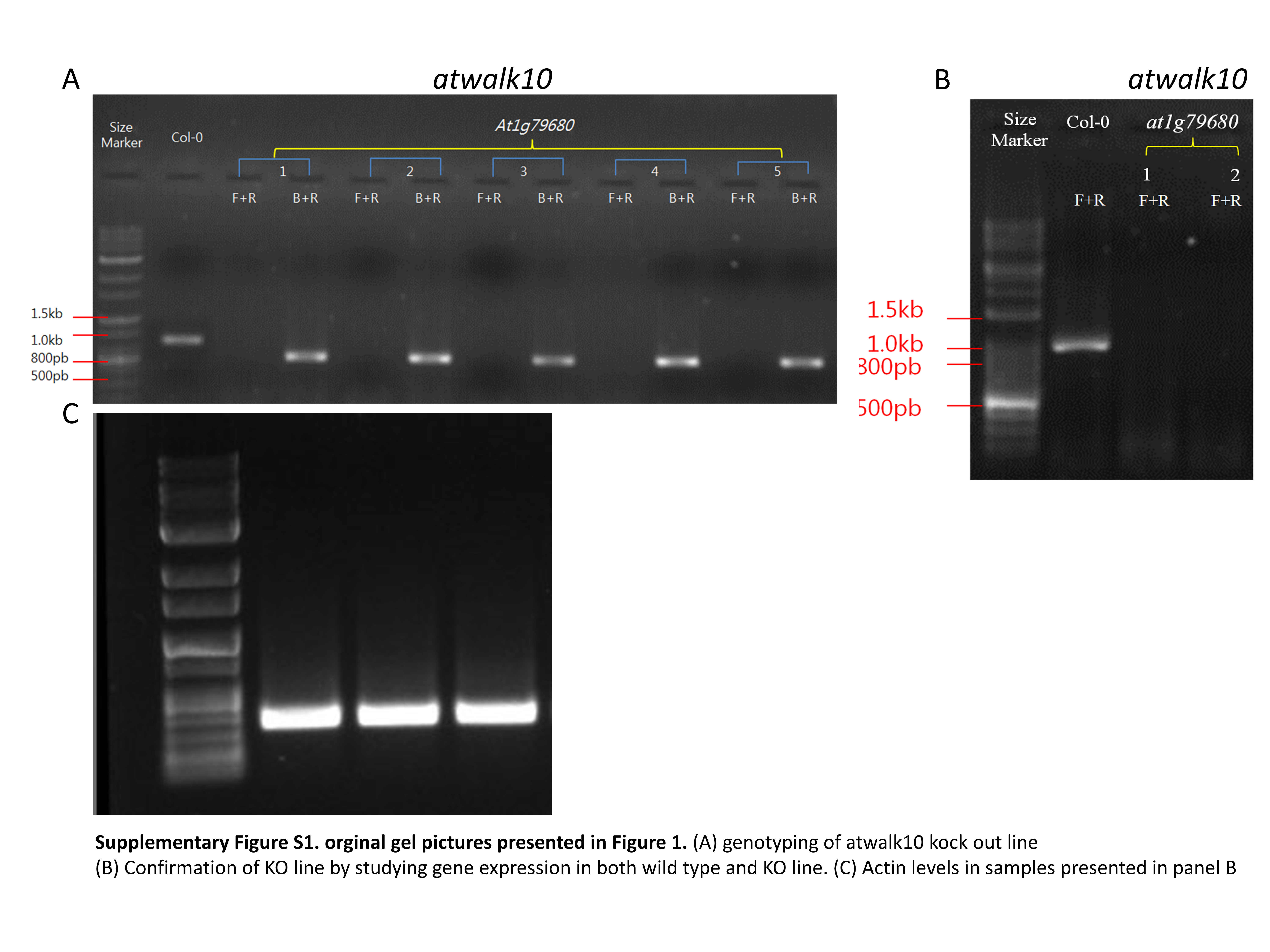

Supplement: Supplemental Information 1 — (A) Genotyping of atwalk10 kock out (KO) line. (B) Confirmation of KO line by studying gene expression in both wild type and KO line. (C) Actin levels in samples presented in (B). [file peerj-07-7383-s001.png]
